# Reflex Pain Following the Extraction of a Tooth

**Published:** 1888-10

**Authors:** V. A. Latham


### Selections.

### Reflex Pain Following the Extraction of a Tooth.

The following case came under my notice whilst a pupil with Dr. Parkinson, England. In July last John R.—age 19—serving the occupation of gardner, and undergoing treatment prior to filling, in the course of which I had the misfortune to leave in the posterior buccal root, a portion of a nerve broach, so small that at the time I was not aware of the accident. A few days later the tooth was removed, when nothing unusual occurred to excite attention. The next day the patient returned, complaining of pain in the vacated tooth socket, and also of pain in the testicles; the latter he felt to a very slight extent when the tooth was extracted. He was told to come on the following day but failed to do so, and shortly afterwards went to the sea side. He returned from Hastings a month after, better, and called at the surgery, when I learned that the pain in the two situations continued for a month after the tooth was removed, and generally appeared at the two places simultaneously. There were no physical alterations in the glands; he had gonorrhœa ten years ago, from which he duly recovered and is now married.

V. A. Latham.

